# Editorial: Protein Degradation Pathways in Parkinson's Disease and Neurodegeneration

**DOI:** 10.3389/fnins.2020.00741

**Published:** 2020-07-28

**Authors:** Mattia Volta, Patrick A. Lewis, Evy Lobbestael

**Affiliations:** ^1^Institute for Biomedicine, Eurac Research, Bolzano, Italy; ^2^Royal Veterinary College, London, United Kingdom; ^3^UCL Queen Square Institute of Neurology, London, United Kingdom; ^4^Laboratory for Neurobiology and Gene Therapy, Department for Neurosciences, KU Leuven, Leuven, Belgium

**Keywords:** neurodegeneration, alpha-synuclein, Parkinson's disease, LRRK2, autophagy, vesicles

The most common neurodegenerative diseases of aging are characterized by intraneuronal accumulations of aggregated proteins that are closely linked to pathogenesis and neuronal loss. The formation of these inclusion bodies has been initially related to the biochemical properties of their main protein components, which shared common fibrillization and aggregation propensity (Ross and Poirier, 2004). Neuropathologically, the identification of the aggregated protein helps the ultimate diagnosis. Thus, alpha-synuclein pathology is classically related to Parkinson's disease (PD), Tau and Amyloid-β to Alzheimer's disease (AD), SOD1, TDP-43, and C9orf72 to Amyotrophic Lateral Sclerosis/Frontotemporal Dementia (ALS/FTD), Huntingtin to Huntington's disease (HD). Interestingly, some of these pathologies overlap in the same clinical entity (e.g., alpha-synuclein pathology in AD). Nevertheless, the etiological role of these inclusions has been questioned and is still a matter of intense debate (Surmeier et al., 2017). Efforts to uncover the mechanistic role of neuropathology have led to important advances in cell biology and neurobiology pointing to the involvement of protein degradation pathways in neurodegeneration, especially the ubiquitin-proteasome system (Tai and Schuman, 2008) and autophagy (Scrivo et al., 2018).

In Parkinson's disease, the study of the autophagy-lysosome pathway has rapidly progressed in recent years. Autophagy is implicated in alpha-synuclein pathology as impairment in this process causes alpha-synuclein accumulation (Obergasteiger et al., 2018). In addition, a number of genes linked to familial PD have been found to play a role in the modulation of autophagy, especially alpha-synuclein itself and leucine-rich repeat kinase 2 (LRRK2) (Manzoni and Lewis, 2013). Thus, the importance of dissecting these molecular and cellular pathways, and understanding the intimate regulation of PD-linked genes are crucially required to understand the pathogenic processes underlying protein aggregation and neurodegeneration.

We have collected a Research Topic centered on protein aggregation, protein degradation and quality control systems with a focus on how causal genes in PD and neurodegenerative diseases are involved in these processes. These articles provide an overview on different aspects of these processes and discuss how they can be involved in pathophysiology.

The review by Albanese et al. discusses the involvement of autophagy in brain aging and the decline in its efficacy during senescence. The decrease in autophagy function is linked to the accumulation of damaged organelles and misfolded proteins, increasing the risk of cellular damage. In neurons, the severity of this dysfunction is further aggravated by their post-mitotic nature and the incapacity of diluting toxic species with subsequent cell divisions, possibly triggering neurodegeneration. Further, the authors explore how the PD-linked gene *LRRK2* is involved in autophagy regulation and how mutations might affect its physiological function. Moreover, they discuss the possible link to LRRK2-related neurodegeneration, which is of importance, given the fact that LRRK2 is a major pharmaceutical target for neuroprotection in PD.

Closely linked to its role in autophagy is the finding that LRRK2 is a mediator of intracellular vesicle trafficking, which is believed to play an important role in PD pathogenesis.

In this context, the review by Ebanks et al. analyses the role of several genes linked to familial PD or associated at genome-wide level in the modulation of vesicle dynamics and the downstream functional consequences. The authors identify key processes of intracellular vesicle trafficking in which PD genes play crucial roles and that might be involved in the early stages of the disease: vesicle fusion, which is particularly relevant for neuronal transmission; endocytosis, with functions spanning from synaptic vesicle recycling to endosomal transport; the *trans* Golgi network, through which the recently elucidated LRRK2-Rab signaling system mediates vesicle recycling; lysosomal function, critically related to protein degradation and proteostasis with immediate consequences for neuropathology. The authors posit that PD could be regarded as a “dysfunction of vesicular trafficking,” with several etiologic agents converging on such mechanisms and allowing to draw a unifying picture of PD pathogenesis.

Consistent with this view, Rivero-Ríos et al. propose a testable working model to study the effect of the LRRK2-Rab axis in the modulation of the endolysosomal system. They discuss the link between endolysosomal dysfunction and alpha-synuclein aggregation, which could be regarded as a common readout, but caused by distinct mechanisms. For example, different cellular mechanisms (e.g., oligomerization, lysosomal impairment, and lipid perturbations) might underlie the same downstream effect, i.e., the accumulation of intracellular aggregates of alpha-synuclein in patients with alpha-synuclein mutations, GBA mutations or idiopathic PD. In the context of LRRK2-PD, aberrant LRRK2-mediated phosphorylation of Rab8a and Rab10 caused by PD-linked mutations in LRRK2 alters the endolysosomal functionality and could lead to neuronal damage in the absence of alpha-synuclein pathology. When such dysfunctions are paired with further lysosomal stress or damage (e.g., mutations in lysosomal genes or environmental agents), the formation of Lewy bodies would be triggered. Indeed, the authors highlight that a proportion of LRRK2 PD cases do not present with alpha-synuclein neuropathology.

The characterization of neuropathology in different forms of parkinsonism is the subject of the Brief Research Report from Mazzetti et al. Here, the authors histologically analyzed human brain samples from patients who have suffered from synucleinopathies (PD and Multiple System Atrophy) and a tauopathy (Progressive Supranuclear Palsy). Specifically, they investigated the localization of the histone deacetylase HDAC6 and its phosphorylated, active form. HDAC6 is unique in its class as it targets cytoplasmic non-histone proteins and mediates aggresome formation. The authors found that both HDAC6 forms localize to pathological alpha-synuclein and Tau inclusions. In addition, HDAC6 is in close vicinity of alpha-synuclein in PD, as evidenced by a Proximity Ligation Assay. This work sheds light on the biology of protein aggregates and opens up the opportunity of further mechanistic studies to uncover novel mechanisms of neuronal pathology.

Lastly, in their Mini Review Llanos-González et al. discuss the possible central role of oxidative stress in the pathogenesis of Alzheimer's disease. In particular, the authors hypothesize that oxidative stress underlies dysfunction in several cellular domains critically involved in the early onset of the disease. The “first responder” to oxidative-redox imbalance are mitochondria, whose damage triggers a cascade of neurotoxic events in the brain. In addition, oxidative stress can severely impact proteostasis, protein quality control and protein degradation. In this respect, the authors explore the link with endoplasmic reticulum-associated degradation, the unfolded protein response and autophagy. In conclusion, the authors also discuss putative therapeutic approaches targeting oxidative stress that could ameliorate the abovementioned cellular processes.

The five articles collected in this Research Topic provide an advanced update on the state-of-the-art of proteostasis in neurodegeneration, with a prominent focus on PD. The articles explore in further details intracellular vesicle trafficking and its dysregulation in PD conditions, drawing mechanistic links that are centered on proteins implicated in familial PD. Detailed clarification of these processes could lead to the elucidation of a unifying pathogenic process, which would have a major impact on the design of novel, effective disease-modifying therapies.

## Author Contributions

MV, PL, and EL equally contributed to the conception, drafting, and revision of this editorial. All authors contributed to the article and approved the submitted version.

## Conflict of Interest

The authors declare that the research was conducted in the absence of any commercial or financial relationships that could be construed as a potential conflict of interest.
